# Task-Based Eating and Drinking Interventions in Animal Models: A Narrative Review of Functional Improvements and Neuromuscular Adaptations in Age-Related Dysphagia

**DOI:** 10.3390/geriatrics9060138

**Published:** 2024-10-22

**Authors:** Tina Hansen, Sabina Mette Staal, Nete Deela Rauhe Harreby, Ulla Andersen, Masumi Takeuchi Holm, Cecillie von Bülow, Eva Ejlersen Wæhrens

**Affiliations:** 1 Physical Medicine & Rehabilitation Research—Copenhagen (PMR-C), Department of Occupational Therapy and Physiotherapy, Copenhagen University Hospital Amager and Hvidovre, Kettegård Allé 30, 2650 Hvidovre, Denmark; sabina.mette.staal@regionh.dk; 2 Occupation-Centered Occupational Therapy, The Parker Institute, Copenhagen University Hospital Bispebjerg and Frederiksberg, Nordre Fasanvej 57, 2000 Frederiksberg, Denmark; ulla.andersen.03@regionh.dk (U.A.); masumi.takeuchi.holm@regionh.dk (M.T.H.); cecilie.von.bulow@regionh.dk (C.v.B.); eva.elisabet.waehrens@regionh.dk (E.E.W.); 3 Department of Health Science and Technology, Faculty of Medicine, Aalborg University, Selma Lagerloefsvej 249, 9260 Aalborg, Denmark; info@ergospecialisten.dk; 4 Occupational Science, User Perspectives and Community-Based Research, Institute of Public Health, University of Southern Denmark, Campusvej 55, 5030 Odense C, Denmark

**Keywords:** animal models, deglutition disorders, sarcopenia, resistance training, endurance training, motor skills, behavior, neuroplasticity, physiological adaptation, occupational therapy

## Abstract

Background/Objectives: Age-related dysphagia involves sarcopenia and nervous system changes affecting ingestion. The ACT-ING program, a novel task-based occupational therapy intervention, has been developed to improve strength, endurance, and ingestive skills using real-world eating and drinking tasks for older adults with age-related dysphagia. This narrative review evaluates the outcomes and neuromuscular adaptations of task-based eating and drinking interventions in aging animal models to inform potential refinements of the ACT-ING program and interpret results from an ongoing proof-of-concept study. Methods: Publications were obtained from PubMed, SCOPUS, CINAHL, and EMBASE, and selected following the PRISMA guideline. Thirteen randomized trials investigated a task-based fluid-licking intervention in rats, combining strength, endurance, and skill training. Results: Results suggested benefits in improving muscle strength, endurance, and swallowing skills in terms of quantity and speed. Although neuromuscular adaptations were less conclusive, the intervention appeared to induce cortical plasticity and increase fatigue-resistant muscle fibers in the involved muscles. Conclusions: While these findings are promising, methodological concerns and potential biases were identified. Therefore, further research is necessary to refine the ACT-ING program, including both clinical studies in humans and preclinical studies in aging animal models that clearly define interventions targeting all aspects of ingestion-related skills within a motor learning and strength training framework.

## 1. Introduction

Participation in eating and drinking, along with other basic activities of daily living tasks, enhances well-being and quality of life [1]. Eating and drinking tasks satisfy nutritional, social, physical, cognitive, and emotional needs, and contribute to cultural and personal identity [2,3]. Therefore, the ability to ingest food and liquids safely and effectively is crucial for older adults [4]. It enables full participation in meals, maintains autonomy in eating habits, and supports independence [5]. Additionally, it prevents complications such as aspiration, malnutrition, anxiety, embarrassment, or social isolation, thereby enhancing overall quality of life [4,5,6].

Ingestion is a complex, goal-directed process requiring coordinated voluntary and reflexive motor control, mediated by cortical, subcortical, and brainstem centers [7,8,9]. Ingestive skills during mastication and swallowing depend on the ability to modulate the timing, force, and coordination of multiple muscles to perform eating and drinking tasks efficiently. This ensures that task performance during eating and drinking can successfully adapt to variations in variables like bolus size and consistency [10,11]. During mastication, movements of the jaw coordinated with the tongue facilitate bolus formation, saliva mixing, and ultimately, the initiation of swallowing [8,10]. In this process, the tongue plays a crucial role [12]. As the bolus undergoes changes in rheological, surface, and particle size characteristics, sensory information is processed by the masticatory and swallowing central pattern generators. Once a threshold is reached, sensory input triggers the swallowing reflex, usually during expiration, with a brief pause for airway protection [7,8,9,10,12].

Advancing age leads to neuromuscular degeneration in the structures responsible for ingestion, a condition known as presbyphagia. Initially, compensatory adaptations mask these changes, but over time, they may lead to age-related dysphagia [4,13]. This condition can manifest as decreased chewing ability, altered swallowing pressure, delayed swallowing response, impaired coordination between breathing and swallowing, and increased aspiration risk [4,14,15]—all linked to sarcopenia, the progressive loss of muscle mass, quality, and function [16]. In the lingual musculature, sarcopenia results in reduced muscle fiber size and number, a shift to slower-twitch muscle fibers, longer contraction times, and decreased muscle force and endurance [17,18]. Additionally, research indicates disruptions in sensory and motor pathways, including delayed sensory processing, reduced brain activation, pharyngeal hypoesthesia, and neuromuscular degeneration [14,19]. Potential bioenergetic changes in muscle function are also suggested but remain underexplored [20].

Several intervention studies on age-related dysphagia focus on increasing lingual muscle strength through isolated non-swallowing exercises [21,22,23,24,25]. However, since both muscular and nervous system changes contribute to age-related dysphagia, it is recommended to apply interventions that directly incorporate performance during eating and drinking tasks and integrate motor learning theory into strength training approaches [26,27]. Overall, interventions for dysphagia are provided by various health professions, with occupational therapists (OTs) in Denmark playing a primary role in dysphagia management [28]. A Danish occupational therapy initiative, the ACT-ING program, was developed to challenge and improve ingestion functions that have deteriorated due to age-related changes, aiming for long-term enhancements in both ingestive skills and physiological function [29].

The ACT-ING program employs an occupation-based approach referring to direct engagement in occupation as the mean of intervention [30]. In this context, ‘occupation’ refers to the performance of everyday tasks, such as eating and drinking, that individuals need, want, or are expected to do, either independently or within family and community settings, to bring meaning and purpose to life [30]. Through the ACT-ING program, OTs collaborate with clients in shared decision making and goal setting, offering graded, purposeful, and meaningful eating and drinking tasks within relevant contexts as a therapeutic medium for enhancing performance and engagement during meals. This is achieved through a standardized 17-level hierarchy of food and liquid consistencies and volumes [29].

The research on the ACT-ING program is guided by the British Medical Research Council’s (MRC) framework for developing, feasibility testing/piloting, evaluating, and implementing complex interventions in an iterative multi-phase process [31]. In the first phase, the ACT-ING program’s theory and expected mechanisms of change were articulated through several literature reviews and the involvement of clinical and academic experts as well as clients [29]. This resulted in a manualized program that integrates a mixed occupational therapy model approach [30] with simultaneous application of a restorative model focusing on remediation of underlying impairments (i.e., strength, coordination, and timing of ingestion-related muscles), an acquisitional model focusing on maintaining and redeveloping ingestive skills (e.g., chewing, manipulating food in the mouth, swallowing by means of the effortful swallowing technique), and a compensatory model (e.g., strategies to enable an appropriate sitting position) [29]. The gradation of the eating and drinking tasks is based on the strength training principle of progressive overload using food and liquid items as external loads in conjunction with the key principles of motor learning theory, such as practice complexity, amount, variability, distribution, schedule, and feedback [26,27,32]. In the second phase, the early feasibility of the ACT-ING program was assessed in a population of older adults with age-related dysphagia. The intervention was delivered 2–3 times per week with self-training during daily meals and continued for an average of eight weeks. The ACT-ING program was largely endorsed in terms of demand, safety, adverse events, tolerance, usability, acceptability, in-therapy engagement, and improvements in swallowing capacity [33]. Currently, a proof-of-concept study is in progress (ClinicalTrials.gov ID NCT05935618).

Non-pharmacological intervention research, like the ACT-ING program, often parallels pharmaceutical trials, requiring significant preparatory research and hypothesis testing [34]. The ACT-ING program aims to promote externally induced, experience-dependent plasticity (EDP) through progressively challenging the ingestion function and appropriate feedback during therapeutic eating and drinking tasks [29]. In this context, the principles of EDP suggest that functional and peripheral physiological improvements associated with learning are accompanied by plasticity in the neural networks responsible for ingestion [26,27,29]. However, clinical studies on the relationship between peripheral physiology, functional outcomes, and central EDP in older adults with age-related ingestion changes are limited, especially in relation to task-based eating and drinking interventions [26]. Understanding the impact of the ACT-ING program on ingestion-related musculature and performance in eating and drinking tasks is crucial, as is exploring its effects on musculoskeletal and nervous system adaptations [26,34,35]. In contrast to clinical studies in humans, preclinical animal studies can offer valuable insights into neuromuscular adaptations at various levels [35,36], which can inform refinement of the delivery methods, optimal dosing, and the theoretical framework of the ACT-ING program, which is in its early phase of development.

This narrative review summarizes current knowledge and insights gained from aging animal models regarding muscular and nervous system adaptations and outcomes related to task-based eating and drinking interventions for age-related changes in ingestion functions. The primary objective is to guide potential refinements of the ACT-ING program and its theoretical foundations, while also providing a basis for interpreting results from the ongoing proof-of-concept study. The research questions addressed are as follows: (1) Which task-based eating and drinking interventions targeting ingestion functions in aging animal models effectively combine strength and skill training? (2) What neuromuscular adaptations and outcomes result from these interventions?

## 2. Materials and Methods

The review was guided by the criteria of the Scale for the Assessment of Narrative Review articles (SANRA) [37] and the Preferred Reporting Items for Systematic Reviews and Meta-Analyses (PRISMA) statement [38].

### 2.1. Identifying Relevant Studies

The literature search was conducted in the following databases from their inception to May 2024 with regular updates until September 2024: PubMed, SCOPUS, CINAHL, and EMBASE. A combination of keywords and indexing terms relevant to three areas (age-related dysphagia, task-based approaches combining skill and strength training of the ingestive function, and animal models) was initially created for PubMed and subsequently adapted to the other databases. The search was complemented with citation searches and inclusive manual searches of references from relevant reviews.

The search string for PubMed was as follows: ((dysphagia) OR (deglutition disorder)) AND ((aging) OR (old) OR (aged)) AND ((exercise) OR (training) OR (rehabilitation) OR (strength) OR (resistance) OR (endurance) OR (behavioral) OR (skill)) AND ((chewing) OR (mastication) OR (licking) OR (swallowing) OR (deglutition) OR (eating) OR (drinking)) AND ((animal) OR (rats)) OR (rat) OR (rodent) OR (mice)) OR (murine)). The search terms ‘rodent’, ‘rat’, and ‘mice’ were included because they are commonly used in animal models for studying dysphagia [36].

### 2.2. Study Selection

The selection of studies was based on the following inclusion criteria: animal models of aging; evaluation of the impact of task-based eating and drinking interventions for improving ingestion functions; intervention outcomes related to strength, endurance, or ingestive skill acquisition; results on adaptations in the musculoskeletal or nervous systems; experimental studies; peer-reviewed; and in English language. Studies were excluded if they investigated animal models with induced disease conditions [36] or did not include old rats. Additionally, studies were excluded if the intervention was not task-based towards ingestion functions, such as wheel running, or if it involved brain and nerve stimulation methods for swallowing recovery (e.g., transcranial magnetic stimulation, transcutaneous electrical nerve stimulation [26]). The first author (T.H.) was responsible for screening title and abstracts of identified records as well as assessing full-text articles for eligibility.

### 2.3. Data Extraction

The following descriptive data were extracted from eligible studies by T.H.: author(s), year of publication, animal species, age, experimental groups, and intervention variables (task, duration, repetitions, and progression). For intervention outcomes, data on achieved strength, endurance, and ingestive skills were extracted. Data on neuromuscular adaptations were extracted, focusing on neurological, morphological, and bioenergetic adaptations [35]. The results are presented narratively for each individual study, and the reported effect directions for individual outcomes are presented visually and are discussed continuously to provide a comprehensive analysis of the findings in relation to the ACT-ING program.

### 2.4. Risk of Bias Assessment

The risk of bias in the included studies was assessed using the SYstematic Review Centre for Laboratory animal Experimentation (SYRCLE) tool [39]. This assessment was independently conducted by three authors: T.H. and S.M.S. assessed seven studies, while T.H. and N.D.R.H. assessed six studies. To ensure consistency in the methodological quality assessments, the authors first jointly evaluated one study. They discussed the interpretation of the signaling questions in the SYRCLE tool to standardize their assessment approach.

## 3. Results

### 3.1. Literature Search

**Figure 1:**
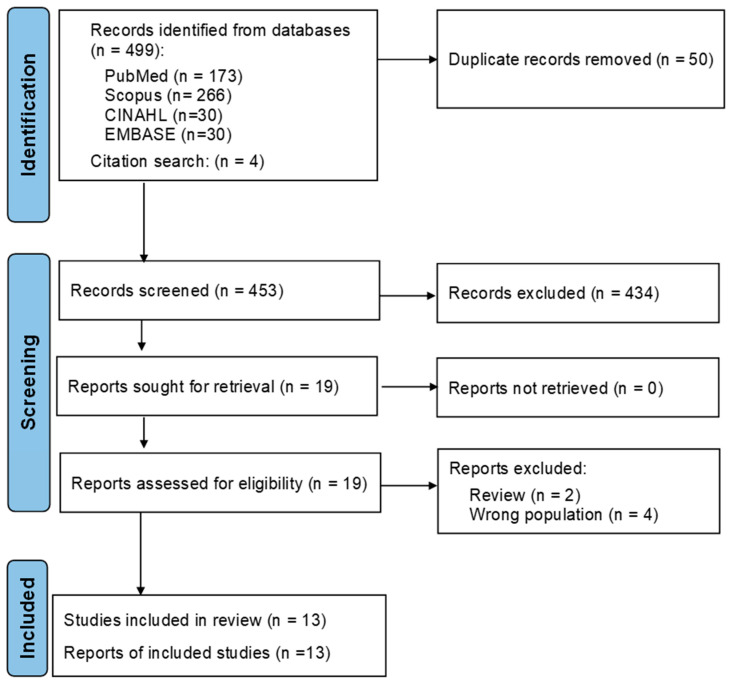
PRISMA 2020 systematic review flowchart of the selection of studies.

As shown in Figure 1, a total of 499 records were identified through the database searches, and four additional records were identified via citation search. After removing duplicates, 453 records remained for screening. Of these, 19 reports were assessed for eligibility. Six reports were excluded for the following reasons: two were review papers [20,40], three did not apply an aging animal model [41,42,43], and one investigated an animal model with an induced disease condition [44]. This resulted in the inclusion of 13 studies [45,46,47,48,49,50,51,52,53,54,55,56,57].

### 3.2. Characteristics of the Included Studies and Intervention Variables

The characteristics of the studies and the intervention variables are presented in Table 1.

#### 3.2.1. Study Characteristics

All thirteen studies were conducted in the USA and were published after 2009. Except for one study [45], they all originate from the same research program at the University of Wisconsin–Madison (UW–Madison). The studies exclusively used male rats. One study [50] cited the potential impact of the estrous cycle in female rats on outcome measures. This exclusion may raise concerns about the generalizability of the findings to female populations and highlights the need for including both sexes in future research to understand potential sex-specific responses [58]. In Guggenmos (2009) [45], rats were considered old at 18 months, while studies from UW–Madison classified rats into three age groups: young adults (YA) aged 6–9 months, middle-aged adults (MA) aged 21–24 months, and old adults (OA) aged 29–33 months [46,47,48,49,50,51,52,53,54,55,56,57]. The classification of 18-month-old rats as old [45] is debatable, suggesting they might be middle-aged instead [59]. In human terms, the rat age categories used in the UW–Madison studies approach 18–24 years (YA), 47–60 years (MA), and 73–82 years (OA). While these categories may capture significant age-related changes, they could miss nuances outside these specific age ranges [59]. The ACT-ING program targets adults above 65 years [29], comparable to a rat age of 26 months [59], a demographic not represented in the included studies. Accordingly, the results from these studies might not entirely apply to the ACT-ING program [59].

All studies were experimental and utilized randomization. Most studies, except for Krekeler (2020) [56]), did not report a sample size calculation, potentially increasing the risk of underpowering and type II errors [60]. Eight studies employed a two-arm design [45,46,47,48,51,52,53,57] while five [49,50,54,55,56] used a multi-arm design. Control groups comprised either an untrained control group or a trained control group that learned the intervention task without resistance to perform a maximal voluntary tongue force (MVTF) test.

#### 3.2.2. Intervention Task

The studies included an intervention that replicate the fluid-licking behavior of rats, involving repetitive tongue and jaw movements, where the rat repeatedly dips the tongue and scoops water into the mouth [61]. In the studies, rats were trained to press their tongues against an 18 mm force-incremented disk, which dispensed a water reward of either 0.05 mL [45] or 0.10 mL [46]. The force-incremented disk was placed in an enclosure with a depressed front panel and a small portal for tongue access. The apparatus was computer-controlled to measure the parameters of the tongue press and deliver the water reward upon meeting preset force criteria. To motivate the rats to perform the task, the intervention model included periods of water restriction [45,46].

The intervention task engages both the extrinsic and intrinsic tongue muscles [46,49,53,56,57]. The extrinsic tongue muscles, including the genioglossus, hyoglossus, styloglossus, and palatoglossus, facilitate bolus formation, placement, and propulsion by protruding, retracting, depressing, and elevating the tongue [9,12]. The intrinsic muscles, such as the superior longitudinal, inferior longitudinal, transverse, and vertical muscles, influence tongue shape and size during bolus formation and propulsion [9,12,18]. All intrinsic and extrinsic tongue muscles, except for the palatoglossus, are innervated by hypoglossal motor neurons (CN XII), while the palatoglossus muscle receives innervation from the vagus nerve (CN X) [9,12].

Overall, it appears that the description of the modeled intervention from UW–Madison has changed over time. Initially, Connor (2009) [46] described the intervention as focusing on progressive resistance tongue exercises to increase protrusive tongue forces, like the Iowa Oral Performance Instrument (IOPI) program for elderly dysphagia patients [22]. Later studies [53,55] redefined the intervention to include both features of endurance and strength training. Cullins (2019) [54] further argued that the intervention also encompasses features of motor learning and skill acquisition.

Connor (2009) suggests that the intervention could be considered a meaningful and functional task for the rats. However, from an occupational therapy perspective, this modeled intervention of fluid-licking behavior may not fully align with an occupation-based approach. Instead, it might be better characterized as a simulated occupation, where task performance is somewhat artificial and includes elements that are not naturally parts of the task [30]. For instance, having rats press their tongues against a force-incremented disk before receiving water introduces an unnatural component to the behavior. In addition, water restriction was used in the intervention model to motivate the rats to perform the fluid-licking task. In contrast, the ACT-ING program prioritizes engagement in ecologically relevant tasks by using real objects, such as food and liquid items, that offer an appropriate level of challenge within a real context, such as the participant’s usual environment for consuming food and liquids. The program also involves participants in the decision-making process regarding the products used, provides therapy at appropriate meal or snack times, and considers the habits and preferences related to meal performance [29,33]. Accordingly, transferring the results from the included studies to humans using the ACT-ING program necessitates careful and critical evaluation.

For future research, it is suggested that behavioral training of rats, like the fluid-licking task, could include an enriched environment without water restrictions, instead using rewards like sugar water, juice, soy milk, or peanut oil [62]. Such enrichment might enhance the active interaction between the animal and its environment and better align with motor learning theory [63]. This approach could also translate better to humans [63,64] and the ACT-ING program [29,33].

No studies with interventions targeting task performances in animals fed a solid food diet were identified. The muscles involved in mastication include the masseter, temporalis, pterygoid, and digastric, which work synchronized with the intrinsic and extrinsic tongue muscles to ensure successful bolus formation and swallowing [8,9,12]. Given the tongue’s crucial role in mastication and the impact of food consistencies on jaw muscle adaptation and cognitive functions [8,65], animal models of aging and interventions addressing both tongue and masticatory muscles are necessary for a comprehensive understanding of combined skill and strength training for ingestion included in the ACT-ING program [29]. However, Ross (2024) [12] argues that mechanisms of tongue movement during mastication and bolus transport may not be the same in different animal models. Rats chew using a bilateral posterior-to-anterior pattern rather than a unilateral lateral-to-medial pattern seen in primates and it is unknown whether similar asymmetrical tongue movements during human mastication are used by rats. For future research, this might suggest inclusion of other species in aging animal models to fully understand their benefits and limitations as models for human ingestion [12].

#### 3.2.3. Duration, Repetitions, and Progression

In the study by Guggenmos (2009) [45], the intervention involved rats licking at a constant pressure of 30 g for 12 repetitions over a period of up to six days. In contrast, the twelve studies conducted at UW–Madison [46,47,48,49,50,51,52,53,54,55,56,57] featured an eight-week intervention duration, with each session lasting 10 min, five days per week. Although the ACT-ING program lasts eight weeks, physiological responses in older humans may differ from those in older rats due to differences in aging between species [59], potentially impacting the application of the results.

The progression of the task required the rats to exert 50% to 80% of their MVTF to receive a water reward, with MVTF initially measured and averaged over three consecutive days (see Table 1). The minimum required repetitions above the threshold, reported in three studies [46,50,57], ranged from 20 to 30 licks per session. Four studies [46,49,53,55] noted an average of 100 tongue presses per session above the reward thresholds. These findings indicate a substantial volume of repetitions, consistent with the general principles of combined strength training and motor learning [27,32]. Guggenmos (2009) [45] implemented a limited amount of practice (12 repetitions) with minimal variability, while Connor (2009) [46] involved extensive practice with progressive thresholds but under constant conditions. The specifics of the distribution and schedule of practice were less detailed. If the fluid-licking task indeed involves motor learning principles, as suggested by Cullins (2019), future animal studies should model interventions more explicitly according to key motor learning principles [62]. This would facilitate a more straightforward transfer of results to humans using the ACT-ING program, in which participants increased their swallowing repetitions per session from an initial 3 to 10 at the beginner level, advancing to an average of 73 repetitions per session. These sessions involved up to 17 different liquid and food items, randomly distributed into 10 sets at the more advanced level [33].

### 3.3. Quality of Studies

**Figure 2:**
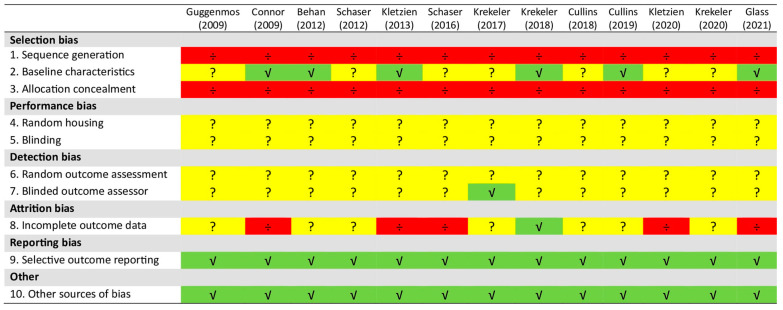
SYRCLE’s risk-of-bias tool assessment of the included studies [45,46,47,48,49,50,51,52,53,54,55,56,57]. Green √ signals ‘yes’, indicating low risk of bias. Red ÷ signals ‘no’, indicating high risk of bias. Yellow ? signals ‘unclear’, indicating unclear risk of bias.

Figure 2 presents the risk of bias assessment for each study.

Although all studies employed randomization, the methodology was either unknown or insufficiently reported, resulting in a high risk of selection bias. Basic animal characteristics were reported in approximately 46% of the studies, with the rats’ body weight used as a covariate in the analyses. Baseline values for the outcomes of interest were frequently lacking, likely due to the nature of the outcome measurements, such as neurotransmitter levels in cranial nerve nuclei.

Performance bias was judged as unclear since no studies reported on the random housing of the rats or blinding of caregivers and investigators. Detection bias was also judged as unclear due to the lack of reports on whether random outcome assessment was undertaken and whether blinding of outcome assessors for each main outcome was ensured. In 27% of the studies, blinding was described for only a few primary outcomes. Reports on missing outcome data were adequately addressed in one study, unclear in seven studies, and inadequately in five studies. Among the studies exhibiting a high risk of attrition bias, not all animals were included in the analysis, and missing values were not accounted for using appropriate imputation methods. Instead, the studies relied on complete case analyses, likely overestimating the treatment effect [60].

Despite the absence of citations to available study protocols, all studies were judged to have a low risk of reporting bias due to coherence between the methods and results sections. Other issues, such as the influence of funders or design-specific risks of bias, were also judged to have a low risk. In the multi-arm studies (see Table 1), all data were presented per group.

It has recently been argued that due to the lack of concordance between guidelines for conducting animal studies and guidelines for their quality assessment, some domains might appear to be at high risk of bias simply because the authors did not document them in their publications, even if those recommendations were followed during the experiment [66]. Therefore, the results might be applicable for the purpose of this narrative review.

### 3.4. Intervention Outcomes

Table 2 displays the effect direction of the reported intervention outcomes, of which all originate from UW–Madison.

#### 3.4.1. Strength and Endurance

Across 11 studies, eight weeks of intervention induced a significant increase in MVTF during fluid licking. This increase was observed both by within-subject analyses [46,47,48,49] and when compared with a trained control group [50,51,53,54,55,56,57]. None of the studies found significant effects related to age.

Krekeler (2020) [56] found that intervention frequencies of 1, 3, or 5 days per week over 8 weeks all led to significant increases in MVTF compared to a trained control group, with the highest frequency resulting in the largest increase. Similarly, Kletzien (2020) [55] reported significant increases in MVTF after two weeks and eight weeks of intervention compared to three days of intervention, likely aligning with the MVTF testing procedure for a trained control group. Additionally, the research group at UW–Madison [50] found that detraining did not eliminate the improved tongue forces in rats performing the fluid-licking task without resistance two or four weeks post-intervention. Tongue forces remained significantly increased compared to baseline, although older rats experienced a relative decline in maintaining post-intervention tongue force levels.

The observed increase in tongue force, even after just a two-week intervention [55] and with fewer weekly sessions [56], may be considered promising in relation to the ACT-ING program. During the feasibility testing of the ACT-ING program, some of the frailest and multimorbid participants showed low adherence to the intervention, as it was periodically interrupted due to advanced diseases or rehospitalizations [33]. The findings of sustained increased tongue force in rats after eight weeks of training with five sessions per week [55] suggest that the intervention could produce long-lasting effects, although some decline might occur in older individuals over time. However, further research is needed to determine the effects of longer detraining periods [67]. Also, it remains unclear whether the gains in tongue force in the rats would persist with fewer weeks and fewer days per week, as implemented in the ACT-ING program. It is also important to note that the outcomes of interventions based on simulated occupations might differ from those incorporating an occupation-based approach [30]. Therefore, further clinical studies in humans are necessary to verify the positive results.

Endurance of the ingestive muscles was not directly measured in any of the included studies. Typically, tongue endurance in humans is assessed by measuring how long individuals can sustain 50% of their maximal tongue pressure using objective tools like the IOPI [68]. Kletzien (2013) [49] indirectly investigated fatigue resistance by repeatedly stimulating the hypoglossal nerve, finding that the intervention was associated with reduced tongue fatigue in older rats compared to an untrained control group. Although the observed increase in fatigue resistance may suggest potential benefits for muscle endurance, future research should include specific endurance metrics to validate these findings and explore their implications for, and translation to, ingestion functions in humans.

#### 3.4.2. Ingestive Skill Acquisition

Ingestive skill acquisition was addressed in three studies. Connor (2009) [46] found that the number of tongue presses within the first two minutes of MVFT testing increased significantly following the eight-week intervention across all age groups. Animal age did not affect the number of tongue presses at baseline or post-intervention. Krekeler (2020) [56] found no intervention effect on bolus volume but did observe significantly increased swallowing speed in the group of older rats receiving the intervention five days per week compared to those undergoing the intervention three days per week. However, since there were significant differences between these two groups at baseline, the authors interpreted the results with caution. Krekeler (2017, 2020) [51,56] found no significant effect on mastication characteristics (i.e., number of bites, intervals between bites, time to eat) of the intervention or the intervention dose.

Translating the findings on skill acquisition to humans, the ACT-ING programs have shown similar increases in swallowing frequency [33]. One hypothesis of the ACT-ING program is that increased swallowing frequency results from learning effortful swallowing within a therapeutic, facilitative environment, which aims to prevent and improve “inactivity-induced” sarcopenia in the ingestive muscles [29]. Connor [46] also suggests that the increased swallowing frequency in the rats could reflect an improvement in task participation due to learning. Given the focus on fluid licking, the lack of effect on masticatory behavior in rats [49,54] is unsurprising. The ACT-ING program adopts a comprehensive approach, incorporating fluid, solid, and semi-solid foods to enhance masticatory and swallowing skills [29,33]. A recent systematic review finds that dietary hardness likely impact positively on behavior, cognition, and brain function in animals as well as humans [65]. Thus, aging animal models with enriched environments, encompassing diverse tastes and textures, could shed light on the ACT-ING program’s putative mechanisms of change and guide its research initiatives.

### 3.5. Neuromuscular Adaptations

Table 3, Table 4 and Table 5 display the effect direction of the reported neuromuscular adaptations.

#### 3.5.1. Neurological Adaptations

Neurological adaptations in response to the intervention were examined in five studies [45,47,48,50,54], focusing on neuroplastic changes and excitability in the sensorimotor cortex representing the muscles of the lips, tongue, and jaw, and synaptic plasticity in the cranial sensorimotor and muscular systems (Table 3).

Neuroplastic changes in the sensorimotor cortex and excitability was investigated in two studies with conflicting results [45,54]. Guggenmos (2009) [45] found no significant intervention effects on the total cortical orolingual movement area. In contrast, Cullins (2019) [54] found a significantly larger tongue motor cortex representation in both trained groups compared to the untrained group, with no significant difference between the two trained groups. There was no correlation between the lingual cortical motor area and MVTF, nor did it differ significantly with age. Regarding motor cortex excitability, Guggenmos (2009) [45] reported significant decreases in current thresholds for evoking orolingual motor responses, while Cullins (2019) [54] found no significant differences in the stimulation current needed to induce tongue or jaw movement.

The findings by Cullins (2019) [54] suggest that learning a novel tongue force skill can induce plasticity in the lingual motor cortex of rats. This might translate to the proposition of EDP for the ACT-ING program, which includes the effortful swallowing technique emphasizing tongue-to-palate pressure [29,33]. However, the conflicting results between Guggenmos (2009) [45] and Cullins (2019) [54] regarding cortical area and motor cortex excitability imply that the intensity and specificity of training within the two programs could influence neuroplastic responses differently. In addition, the conflicting results could stem from differences in the rat-age and the assessment and analysis of orolingual motor area as well as an unclear/high risks of bias, warranting cautious interpretation.

Synaptic plasticity was studied in the hypoglossal and ambiguous nuclei [47,48,50] as well as in the genioglossus and thyroarytenoid muscles [50] by examining concentrations of either neurotransmitters (which facilitate signal transmission between neurons), or neurotrophins (which promote neuron growth and survival) and their specific receptors. The studies indicated age-dependent neuroplastic responses in the hypoglossal nucleus. Behan (2012) [47] found an intervention effect on the neurotransmitter serotonin (5HT) concentration only in young rats, specifically in regions controlling protrusion muscles, with no significant correlations between MVTF and 5HT at any age. Schaser (2012, 2016) [47,50] found no significant intervention effect on the expression of the tropomyosin receptor kinase B (TrkB) for the brain-derived neurotrophic factor (BDNF) in the hypoglossal and ambiguous nuclei or the genioglossus and thyroarytenoid muscles at any age. Schaser (2016) [50] also reported no effect on neurotrophin NT4 concentrations across age groups. However, BDNF concentration results were conflicting: Schaser (2012) [48] observed a significant increase in young rats, while Schaser (2016) [50] found a higher BDNF concentration in old, exercised rats compared to old controls, suggesting an intervention effect in older rats.

These findings of no overt effect on synaptic plasticity in the cranial sensorimotor and muscular systems might be explained by potential under-powering, an unclear/high bias risk, or the included rat strain (i.e., Fischer 344 Brown Norway). It has been suggested that despite notable functional decline of the tongue muscle, neurogenic influences on sarcopenia seem less prominent in the F344 Brown Norway strain compared to other strains [18]. However, learning induces the formation of new synapses in addition to changes of existing synapse strength; it is, therefore, difficult to use the results to inform refinements of the ACT-ING program and its theoretical framework.

#### 3.5.2. Muscle Morphological Adaptations

Muscle morphological adaptations to the 8-week intervention were studied in six UW–Madison studies [46,49,52,53,56,57], focusing on the cross-sectional area (CSA), muscle fiber composition, and muscle contraction properties (Table 4).

No significant CSA increases were observed in the genioglossus [46,56], intrinsic tongue muscles [53], or posterior digastric muscles [52]. However, Connor (2009) [46] noted a trend towards an increased CSA in the genioglossus with high variability, and Krekeler (2018) [52] found decreased temporalis muscle fiber size in older rats. These findings align the effect of resistance training on limb strength gains in old populations [67,69]. In a meta-analysis, Straight (2020) [69] observed that the hypertrophic response to resistance training decreases with advancing age, regardless of the intervention duration. In this context it might not be expected that the ACT-ING program [29,33] will lead to muscular hypertrophy increases in muscle size across the ingestive muscle groups.

Muscle fiber composition was assessed via myosin heavy chain (MHC) isoforms: type I (slow/oxidative and fatigue-resistant), type IIa (fast/oxidative and less fatigue-resistant than type I), type IIx (fast and intermediate), and type IIb (fast/glycolytic and fatigable) [49,53,56,57]. Kletzien (2013) [49] reported increased MHC type I and IIa and decreased type IIb in the genioglossus, while Krekeler (2020) [56] and Glass (2021) [57] found no significant changes. Cullins (2018) [53] noted regional shifts in the intrinsic tongue muscles but no age differences. Similarly, Krekeler (2018) [56] found no significant changes in MHC isoforms in the posterior digastric or temporalis muscles. These findings might suggest that the intervention increases fatigue-resistant muscle fibers in the ingestive muscles directly involved in fluid licking. Thus, it might be expected that the ACT-ING program will lead to an increase in the number of fatigue-resistant muscle fibers of the muscles involved in drinking and swallowing, thereby enhancing the task performance of older adults during daily meals.

The studies on muscle contraction properties for the genioglossus [49], hyoglossus, and styloglossus [57] muscles found no significant effects on contraction forces or temporal properties. Given that the 8-week intervention did not significantly affect the CSA of the genioglossus, it is important to note that the CSA partly determines the number of cross-bridges available to bind in parallel. In addition, intrinsic contractile function appears to be relatively well-preserved in older men and women [70]. In this context, it might not be expected that the ACT-ING program [29,33] will lead to changes in muscle contraction properties of the target group.

#### 3.5.3. Bioenergetic Adaptations

Bioenergetic adaptations following the intervention were explored in two UW–Madison studies focusing on muscle metabolism and regeneration (Table 5).

Krekeler (2020) [56] investigated mitochondrial content and function in the genioglossus muscle, finding no significant changes in metabolism post-intervention. Meanwhile, Kletzien (2020) [55] studied muscle regeneration in the genioglossus, intrinsic tongue muscles, styloglossus, and hyoglossus, assessing satellite cell content, activation, and differentiation using genetic markers. Kletzien (2020) [55] found no impact on muscle regeneration in the genioglossus or intrinsic tongue muscles. However, Kletzien (2020) [55] noted significantly elevated genetic marker levels in the styloglossus and hyoglossus muscles. Moreover, a significant correlation was found between genetic marker levels across all tongue muscles and MVTF.

Although the fluid-licking task did not significantly alter mitochondrial function in the genioglossus or stimulate satellite cell activity in the genioglossus and intrinsic tongue muscles, it did affect genetic markers associated with muscle regeneration in other tongue muscles. These findings suggest nuanced regional adaptations within the tongue musculature and highlight the importance of considering multiple aspects of muscle biology when evaluating intervention outcomes [12]. In this context, the ACT-ING program [29,33] may not directly enhance mitochondrial metabolism or promote muscle regeneration in all tongue muscles. In contrast, it could influence satellite cell activity and genetic markers related to muscle recovery and strength in specific muscles, such as the styloglossus and hyoglossus. Additionally, the correlation between genetic markers and MVTF across all muscles suggests potential benefits for overall tongue muscle function. However, the presence of unclear/high risk of bias necessitates cautious interpretation and future research.

## 4. Methodological Consideration

This narrative review summarized and discussed current knowledge from animal models on task-based eating and drinking interventions for age-related ingestion changes and examined potential outcomes and adaptations within the muscular and nervous systems. However, some methodological consideration needs to be addressed.

It is crucial to recognize the anatomical and physiological differences between animals and humans [12]. These differences may limit the direct applicability of findings from animal models to human clinical practice, underscoring the need for careful interpretation and further validation in human studies [35,36,63].

Overall, the literature review was comprehensive, the inclusion criteria were clearly defined, and the risk of bias assessment was based on a recognized approach. However, one major limitation of this review is that the literature search, study selection, and data extraction was exclusively undertaken by the first author. Also restricting studies to those published in English may introduce publication bias, and excluding studies with disease-induced models that used task-based approaches could have omitted relevant findings on outcomes and neuromuscular adaptations. While rats are commonly used and have similarities to human anatomy, differences in size, posture, biomechanical and cortical control of tongue movements during chewing and swallowing compared to humans may affect the generalizability of findings to clinical applications [9,12]. In addition, the unclear and high risk of bias identified across studies could affect the reliability and validity of the synthesized results and may limit the strength of conclusions drawn from the included studies [66]. Future research efforts should prioritize addressing these methodological shortcomings to improve the reliability and applicability of findings in advancing our understanding of neuromuscular adaptations and therapeutic interventions such as the ACT-ING program.

## 5. Conclusions

Only one type of task-based eating and drinking intervention addressing age-related changes in ingestion function was identified and investigated across 13 animal studies. This intervention, which mimics the fluid-licking performance of rats, integrates strength, endurance, and ingestive skill-acquisition. Although transferring the results from the included studies to humans using the ACT-ING program necessitates careful and critical evaluation, the results suggest potential benefits. Tongue muscle strength, endurance, and skill acquisition were enhanced, as evidenced by improvements in the number and speed of swallows. These benefits were observed with intervention frequencies of five days per week over two to eight weeks and one, three, or five days per week over eight weeks. This finding aligns positively with the ACT-ING program, which operates two to three days per week over eight weeks, targeting a frail population often facing challenges in adherence due to advanced diseases or rehospitalizations. Although the effects on neuromuscular adaptations from the fluid-licking task in rats remain unclear, it appears that cortical plasticity was induced and that fatigue-resistant muscle fibers in the muscles directly involved in fluid licking increased. These findings are promising for the ACT-ING program, which aims to induce experience-dependent plasticity and enhance the ability of older frail adults to sustain engagement in meals. However, the risk of bias assessment revealed that 8 out of 10 entries indicated an unclear or high risk, particularly in selection, performance, detection, and attrition biases. To deepen the understanding of the ACT-ING program’s efficacy in treating age-related dysphagia, further research is required before refining the program. This includes both clinical studies in humans and preclinical studies in aging animal models. Such studies should clearly delineate interventions targeting all aspects of ingestion-related skills within a motor learning and strength training framework.

## Figures and Tables

**Table 1:** Characteristics of included studies and intervention variables.

Publication	Aim ^§^	Animal/Sex Age in Mos. (Age-Group)	Design (Sample Size)	Task Dose	Repetitions Per Session	Progression
Guggenmos (2009) [45]	Assess task-specific changes in orofacial cortical motor areas and thresholds for orolingual movements.	Rat */male 18 (OA)	Randomization TE (OA = 6) UC (OA = 6)	Fluid licking 4 wk, 6 min/d	12	10–30 g, with 30 g constant for 4–6 d.
Connor (2009) [46]	Develop a model for tongue exercise as therapy, assessing force and muscle fiber CSA changes across age groups.	Rat ^#^/male 6 (YA) 21 (MA) 29 (OA)	Randomization TE (YA = 8/MA = 8/OA = 7) UC (YA = 8/MA = 8/OA = 7)	Fluid licking 8 wk, 10 min, 5 d/wk	Min 30. Average: 93–108.	% MVFT Wk 1/2: 50% Wk 3/4: 60% Wk 5/6: 70% Wk 7/8: 80%.
Behan (2012) [47]	Investigate if tongue exercise modifies age-related serotonin changes in the hypoglossal nucleus.	Rat ^#^/male 9–10 (YA) 24–25 (MA) 32–33 (OA)	Randomization TE (YA = 8/MA = 8/OA = 8) UC (YA = 8/MA = 8/OA = 8)	Fluid licking 8 wk, 5 d/wk	NR	From Conor (2009) [46].
Schaser (2012) [48]	Determine the effects of tongue exercise on the HN.	Equals Behan (2012) [47]	Randomization Equals Behan (2012) [47].	Fluid licking 8 wk, 5 d/wk	NR	From Conor (2009) [46].
Kletzien (2013) [49]	Compare tongue exercise and treadmill running effects across age groups in rats.	Rat ^#^/male 9 (YA) 24 (MA) 32 (OA)	Randomization TE (n = 36) UC (n = 31) TR (n = 35)	Fluid licking 8 wk, 10 min, 5 d/wk	Average: 73.5–100.6.	From Conor (2009) [46].
Schaser (2016) [50]	Explore tongue exercise impacts on force, vocalization, and neuroplasticity in voice and swallowing muscles	Rat ^#^/male 9 (YA) 32 (OA)	Randomization TE (YA = 10/OA = 10) DG2 (YA = 10/OA = 10) DG4 (YA = 10/OA = 10) TC (YA = 10/OA = 10)	Fluid licking 8 wk, 10 min, 5 d/wk	Min. 20.	From Conor (2009) [46].
Krekeler (2017) [51]	Determine the effects of tongue exercise on mastication in young adult and old rats.	Rat ^#^/male 9 (YA) 32 (OA)	Randomization TE (YA = 9/OA = 9) TC (YA = 8/OA = 8)	Fluid licking 8 wk, 10 min, 5 d/wk	NR	From Conor (2009) [46].
Krekeler (2018) [52]	Analyze the impact of tongue exercise on the biochemistry and morphology of mastication muscles.	Equals Krekeler (2017) [51]	Randomization Equals Krekeler (2017) [51].	Fluid licking 8 wk, 10 min, 5 d/wk	NR	From Conor (2009) [46].
Cullins (2018) [53]	Explore the impact of exercise on the tongue muscles.	Rat ^#^/male 9 (YA) 32 (OA)	Randomization TE (YA = 8/OA = 8) TC (YA = 8/OA = 8)	Fluid licking 8 wks 5 d/wk	Average: 141	From Conor (2009) [46].
Cullins (2019) [54]	Investigate neural mechanisms underlying age-related swallowing decline and lingual strength.	Rat ^#^/male 9 (YA) 32 (OA)	Randomization TE (YA = 9/OA = 9) UC (YA = 7/OA = 8) TC (YA = 9/OA = 9)	Fluid licking 8 wk, 10 min, 5 d/wk	NR	From Conor (2009) [46].
Kletzien (2020) [55]	Investigate tongue exercise’s ability to enhance satellite cell regeneration in aging tongue muscles.	Rat ^#^/male 7–9 (YA) 30–32 (OA)	Randomization TC(t1) (YA = 10–14/OA = 10–14) TE(t2) (YA = 10–14/OA = 10–14) TE(t3) (YA = 10–14/OA = 10–14) UC (YA = 10–14/OA = 10–14)	Fluid licking t1: 3 d t2: 2 wk t3: 8 wk, 10 min, 5 d/wk	NR	From Conor 2009 [46].
Krekeler (2020) [56]	Assess how exercise frequency affects tongue muscle properties and swallowing.	Rat ^#^/male 9 (YA) 32 (OA)	Randomization TE1 (YA = 10/OA = 11) TE3 (YA = 10/OA = 10) TE5 (YA = 10/OA = 10) TC (YA = 10/OA = 10)	Fluid licking 8 wk TE1: 1 d/wk TE3: 3 d/wk TE5: 5 d/wk TC: 5 d/wk	Approx. 100.	From Conor (2009) [46].
Glass (2021) [57]	Study protrusive tongue exercise’s impact on retrusive tongue muscle biology.	Rat ^#^/male 9 (YA) 24 (MA) 32 (OA)	Randomization TE (YA = 13/MA = 12/OA = 13) TC (YA = 11/MA = 11/OA = 13)	Fluid licking 8 wk, 10 min, 5 d/wk	Min. 20.	From Conor (2009) [46].

Abbreviations: Approx, approximately; CSA, cross-sectional area; d, day(s); DG2, detraining group 2 wk.; DG4, detraining group 4 wk.; HN, hypoglossal nucleus; MA, middle-aged adult; min, minutes; mos., months; Min, minimum; MVTF, maximum voluntary tongue force to obtain a water reward; NR, not reported; OA, old adults; TC, trained control group performed MVTF testing, and was allowed brief water licking without resistance; TE, trained exercise group underwent the intervention and MVTF testing; TE1, one day exercise per week; T3, three days exercise per week; TE5, five days exercise per week; TR, treadmill running; t1, t2, t3, three timepoints; UC, untrained control group, naive to the intervention task; wk, week(s); YA, young adult. Symbols: ^§^ Aim abbreviated; * Rat strain: Sprague-Dawley rats; ^#^ Rat strain: Fischer 344 Brown Norway rats.

**Table 2:** Intervention outcomes of an 8-week fluid-licking intervention.

Publications	Analysis	Strength	Endurance	Skill Acquisition
Connor (2009) [46]	WSA	MVTF ▴	NA	Number of tongue presses ▴
Behan (2012) [47]	WSA	MVTF ▴	NA	NA
Schaser (2012) [48]	WSA	MVTF ▴	NA	NA
Kletzien (2013) [49]	WSA	MVTF ▴	Fatigue ▾	NA
Schaser (2016) [50]	BSA	MVTF ▴	NA	NA
Krekeler (2017) [51]	BSA	MVTF ▴	NA	Mastication characteristics ↔
Cullins (2018) [53]	BSA	MVTF ▴	NA	NA
Cullins (2019) [54]	BSA	MVTF ▴	NA	NA
Kletzien (2020) [55]	BSA	MVTF ▴	NA	NA
Krekeler (2020) [56]	BSA	MVTF ▴	NA	Mastication rate ↔
				Swallowing speed ▴
				Bolus volume ↔
Glass (2021) [57]	BSA	MVTF ▴	NA	NA

Abbreviations: BSA, between subject analysis; MVTF, maximal voluntary tongue force during voluntary drinking; NA, not addressed in the study; WSA, within subject analysis. Effect direction: upward arrow = increase in outcome, downward arrow = decrease in outcome, sideways arrow = no change in outcome. Statistical significance: black arrow *p* < 0.05; open arrow *p* > 0.05.

**Table 3:** Neurological adaptations in response to a fluid-licking intervention.

Publication	Neurological Adaptations	Results
Guggenmos (2009) [45]	Cortical area: orolingual	↔
	Excitability of MC	▴
Cullins (2019) [54]	Cortical area: tongue	▴
	Cortical area: jaw	↔
	Excitability of MC	↔
Behan (2012) [47]	Neurotransmitter: 5HT in HN	↔
Schaser (2012) [48]	Neurotrophin: BDNF in HN	↔
	Receptor: TrkB in HN	↔
Schaser (2016) [50]	Neurotrophin: BDNF in HN	▴
	Neurotrophin: BDNF in NA, GG, TA	↔
	Neurotrophin: NT4 in HN, NA, GG, TA	↔
	Recptor: TrkB in HN, NA, GG, TA	↔

Abbreviations: 5HT, excitatory serotonergic (5HT) input; BDNF, brain-derived neurotrophic factor; GG, genioglossus muscle; HN, hypoglossal nucleus; MC, motor cortex; NA, nucleus ambiguous; NT4, neurotrophin 4; PD, posterior belly of digastric muscle; Post, posterior; SG, styloglossus muscle; SL; superior longitudinal muscle; TA; thyroarytenoid muscle; TrkB, tropomyosin receptor kinase B (receptor for BDNF). Effect direction: upward arrow = increase in outcome, sideways arrow = no change in outcome. Statistical significance: black arrow *p* < 0.05; open arrow *p* > 0.05.

**Table 4:** Morphological adaptations in response to a fluid-licking intervention.

Publication	Morphological Adaptations	Results
Connor (2009) [46]	Muscle fiber CSA in GG.	↑
	Variability of the CSA in GG.	▴
Kletzien (2013) [49]	Muscle fiber composition in GG.	I ▴ IIa ↓ IIx ↑ IIb ↓
	Contractile properties of GG.	↔
Krekeler (2018) [52]	Muscle fiber CSA in PD.	↔
	Variability of the CSA in PD.	↔
	Muscle fiber CSA in Tm.	▾
	Variability of the CSA in Tm	▴
	Muscle fiber composition in DP	I, IIa, IIx, IIb ↔
	Fiber composition in Tm	I, IIa, IIx, IIb ↔
Cullins (2018) [53]	Muscle fiber CSA in IT	↔
	Muscle fiber composition in V	IIa, IIx, IIb ↔
	Muscle fiber composition in T (Ant.)	IIa ↓ IIx ▾ IIb ↑
	Muscle fiber composition in T (Mid.)	IIa ▴ IIx ↓ IIb ↓
	Muscle fiber composition in SL (Mid.)	IIa ↓ IIx ▾ IIb ↑
	Muscle fiber composition in IL (Post.)	IIa ▴ IIx ↓ IIb ▾
Krekeler (2020) [56]	Muscle fiber CSA in GG	↔
	Fiber composition in GG	I, IIa, IIx, IIb ↔
Glass (2021) [57]	Muscle fiber composition in HG	I, IIa, IIx, IIb ↔
	Muscle fiber composition in SG	I, IIa, IIx, IIb ↔
	Contractile properties of HG	↔
	Contractile properties of SG	↔

Abbreviations: Ant, anterior; CSA, cross-sectional area; IL, inferior longitudinal muscle; GG, genioglossus muscle; HG, hyoglossus muscle; IT, intrinsic tongue muscles; Mid, middle; PD, posterior belly of digastric muscle; Post, posterior; SG, styloglossus muscle; SL; superior longitudinal muscle; T, transverse muscle; Tm, temporalis muscle; V, vertical muscle. Effect direction: upward arrow = increase in outcome, downward arrow = decrease in outcome, sideways arrow = no change in outcome. Statistical significance: black arrow *p* < 0.05; open arrow *p* > 0.05.

**Table 5:** Bioenergetic adaptations in response to a fluid-licking intervention.

Krekeler (2020) [56]	Mitochondrial content/function in GG	↔
Kletzien (2020) [55]	Muscle regeneration in GG	↔
	Muscle regeneration in IT	↔
	Muscle regeneration in SG + HG	▴

Abbreviations: GG, genioglossus muscle; HG, hyoglossus muscle; IT, intrinsic tongue muscles; SG, styloglossus muscle. Effect direction: upward arrow = increase in outcome, sideways arrow = no change in outcome. Statistical significance: black arrow *p* < 0.05; open arrow *p* > 0.05.

## Data Availability

No new data were created or analyzed in this study. Data sharing is not applicable to this article.
